# Use of expert elicitation in the field of occupational hygiene: Comparison of expert and observed data distributions

**DOI:** 10.1371/journal.pone.0269704

**Published:** 2022-06-08

**Authors:** David Michael Lowry, Lin Fritschi, Benjamin J. Mullins, Rebecca A. O’Leary

**Affiliations:** 1 School of Population Health, Curtin University, Perth, Western Australia; 2 Department of Primary Industries and Regional Development, Perth, Western Australia; Harvard School of Public Health, UNITED STATES

## Abstract

The concept of professional judgement underpins the way in which an occupational hygienist assesses an exposure problem. Despite the importance placed on professional judgement in the discipline, a method of assessment to characterise accuracy has not been available. In this paper, we assess the professional judgement of four occupational hygienists (‘experts’) when completing exposure assessments on a range of airborne contaminants across a number of job roles within a surface mining environment in the Pilbara region of Western Australia. The job roles assessed were project driller, mobile equipment operator, fixed plant maintainer, and drill and blast operator. The contaminants of interest were respirable crystalline silica, respirable dust, and inhalable dust. The novel approach of eliciting exposure estimates focusing on contaminant concentration and attribution of an exposure standard estimate was used. The majority of the elicited values were highly skewed; therefore, a scaled Beta distribution were fitted. These elicited fitted distributions were then compared to measured data distributions, the results of which had been collected as part of an occupational hygiene program assessing full-shift exposures to the same contaminants and job roles assessed by the experts. Our findings suggest that the participating experts within this study tended to overestimate exposures. In addition, the participating experts were more accurate at estimating percentage of an exposure standard than contaminant concentration. We demonstrate that this elicitation approach and the encoding methodology contained within can be applied to assess accuracy of exposure judgements which will impact on worker protection and occupational health outcomes.

## Introduction

Accurate exposure judgments are the foundation of efficient and effective exposure management. The principal goal of the occupational hygiene professional is to protect all workers by reducing workplace health risks to as low as reasonably practicable. Of paramount importance is understanding worker exposure through direct measurement, but limited resources usually mean that hygienists need to apply a level of ‘professional judgement’, that is, the determination of whether an occupational exposure is acceptable based on limited information [1]. Qualitative exposure judgments based on subjective professional judgement form the foundation upon which most exposure assessments are based, and their accuracy is essential in ensuring appropriate risk management outcomes [2–4]. Professional judgement is considered a tool in the toolkit of the hygienist alongside the series of statistical parameters and analyses (i.e., sample size calculation, result aggregation, conformance assessment based on decision statistics) that are useful for describing exposure profiles in a quantitative fashion. However, the circumstances under which professional judgement is prescribed and understanding who can adequately dispense this expertise is still a topic for which ambiguity exists. Although the notion of professional judgement is generally accepted in the discipline of occupational hygiene, the definition is open to interpretation. Professional judgement may be exhibited through the application of knowledge, skills and experience in a way that is informed by professional standards, laws and ethical principles to develop an opinion or decision.

Any strategy where occupational hygienists make exposure judgments without adequate information or data has the potential to introduce inaccuracy and bias which could leave workers unprotected [1]. The process of making exposure judgments with inadequate information has sometimes been referred to as the ‘art’ of professional judgment. Expert elicitation is the process of retrieving and quantifying expert knowledge in a particular domain [5]. The use of expert elicitation helps to introduce a structure for validation to make the process more transparent and effective [1, 3, 4].

### Accuracy of professional judgement

The application of professional judgement is an integral part of a hygienist’s role and can determine whether resources applied to risk controls, respiratory protection, health surveillance and awareness programs effectively protect workers. Several studies have been published on the accuracy of professional judgment when completing exposure assessments in the field of occupational hygiene [6–11]. Some [3, 4, 12] involved a desktop assessment where qualitative task information and quantitative sampling data were provided while others relied on a walkthrough assessment where direct task observation was employed. The quantitative studies demonstrated that the accuracy of exposure judgments made by hygienists when monitoring data are available is low (<50% correct judgments) but still better than chance (25%) [3, 12]. A number of factors relating to experience, training, certification, and educational level were significant predictors of judgment accuracy [3, 12]. Findings from the walkthrough assessment approach where monitoring data were not available indicated the accuracy of exposure judgments made by hygienists (30% correct judgements) was not much different from chance (25%) [3, 12] and underestimation bias was also present.

Most exposure judgments made by hygienists are qualitative and can often be the determining factor as to whether any measurements should be made. Low accuracy of these judgments can therefore lead to incorrect follow-up activities, which may place workers at risk. Recent findings suggest that the understanding of how workplace factors affect exposure needs to be significantly improved among practitioners [7, 13] and that low accuracy in exposure assessment could be due to occupational hygienists receiving little formal training on how to conduct a basic exposure characterisation [14]. If this step of the exposure assessment is not conducted in a systematic way the hygienist may not investigate the exposure that presents the highest exposure potential with enough detail, leading to low judgment accuracy [14].

### Cognitive biases and heuristics

A principal factor relating to the accuracy of professional judgement may be that of cognitive biases associated with the understanding of skewed lognormal distributions which are common in industrial hygiene data [3, 15]. When reviewing these distributions, mental shortcuts, known as heuristics, are often used which can lead to errors in judgment and introduce bias., There are three types of heuristics: availability, representativeness, and anchoring and adjustment [16, 17]. The availability heuristic reflects the tendency to equate the probability of an event with the ease with which an occurrence can be retrieved from our memory [16, 17]. For example, a hygienist may recall a family member or acquaintance who has suffered an asbestos-related disease, and thus may judge severity of asbestos exposure on the experiences of those around them. This may lead to a discounting of offsetting information, especially when such data conflict with easily recalled personal experience [18]. The degree to which a person’s experiences and memory matches the true frequency determines whether these judgments are accurate. The representativeness heuristic reflects the assignment of an object or event to a specific group or class of events. If the decision maker lacks relevant experience, a surrogate (and less relevant) memory may be used, such as using a normal distribution rather than a skewed log-normal distribution. The anchoring and adjustment heuristic is a strategy for estimating uncertain quantities [16, 17]. When trying to determine the correct value, our minds ‘anchor’ on a value, and then adjust to accommodate additional information. The degree to which our final answer is anchored to the initial value can be influenced by many factors resulting in incorrect conclusions.

Despite these drawbacks, the use of expert knowledge in decision making has been gaining traction [19–21]. and have been shown to improve decision making across a broad range of disciplines, including psychology [8, 22], drug delivery and development [23], transdermal delivery and toxicity [24] environmental exposure assessment [25], habitats of rare species [26] and aggregate exposure assessment [27]. These approaches are particularly useful in areas where a traditional approach of using measured data may be problematic, such as occupational exposure assessment.

The main purpose of this study was to use expert elicitation to assess the professional judgement of a group of occupational hygienists (‘experts’) when completing exposure assessments on a range of airborne contaminants across a number of job roles within a surface mining environment. To achieve this, we assessed professional judgment accuracy by comparing expert judgements with quantitative exposure monitoring data.

## Methods

An expert is commonly defined as someone with comprehensive and authoritative knowledge in an area not possessed by most people [28]. In the discipline of occupational hygiene in Australia, practitioners who attain the status of Certified Occupational Hygienist (COH) are recognised as experts in their field, and this was a prerequisite for participation in our study. The expert group consisted of four COHs, who all had working knowledge of the mining industry (currently employed in mining industry with a minimum of 15 years’ experience working in a mining environment), the job roles, the contaminants of interest and the units and scales to be used in the elicitation process [29]. Notification of recruitment for the study was distributed through email with four of ten experts self-selecting into the study. Informed consent was obtained prior to participation. Two of the participating experts were located in Perth, Western Australia and two experts were located in Brisbane, Queensland. All four experts held a bachelor’s degree, with three of the experts holding a master’s degree and one holding a doctorate. All participating experts were male with the age range being 35–56 years. All data analysis was conducted by the authors in Perth, Western Australia.

### Expert elicitation framework

One of the most important aspects of an elicitation protocol is the choice of summary statistics used to describe the distribution and the order in which these statistics are elicited [30–32]. These summary statistics need to be meaningful to the experts, especially when the experts have limited statistical and probability knowledge [33]. We created a protocol for elicitation which had the experts estimating point estimate values in the following sequence (i) lowest expected value (lowest value that would not surprise the expert), (ii) highest expected value (highest value that would not surprise the expert), and (iii) most common expected value (estimated most likely value that would lie between estimated ‘lowest’ and ‘highest’ values). The exact wording “most common” was employed to make certain that the elicited parameter matched to the model (mode of the distribution). The experts were asked to estimate both concentration and percentage of relevant occupational exposure limit (OEL). The elicitation steps, parameter descriptors, elicitation tool (Excel document) and relevant exposure limits were provided to the experts by email (refer to elicitation tool in the S1 Data).

### Measured data

The measured data were collected in the form of full-shift, personal samples for the following job roles—project driller, mobile equipment operator, fixed plant maintainer, and drill and blast operator (Table 1). Locations for sampling included six iron ore mines located in the Pilbara region of Western Australia. The contaminants of interest were respirable crystalline silica, respirable dust, and inhalable dust. Personal samples were collected and analysed as per the applicable Australian Standard for each agent of interest, these being AS 2985–2009: *Workplace atmospheres–Method for sampling and gravimetric determination of respirable dust* and AS 3640–2009: *Workplace atmospheres–Method for sampling and gravimetric determination of inhalable dust*. Workers were selected randomly whenever possible using a random number table generated through the use of the RAND function in Excel. Equipment used to conduct the air sampling included an SKC AirChek 2000 pump with flexible tubing to 25mm diameter filters supported by a PVC cyclone or IOM sample head, depending on the agent to be measured. The designated flow rate for all samples collected was as per Australian Standards AS 2985:2009 (respirable fractions) and AS 3640:2009 (inhalable fractions) and was adjusted accurately using a calibrated flow meter (Defender 520 Model). All efforts were made to ensure calibration equipment and technique was of such accuracy that the flow rate was measured to within ±5%. Any samples that did not meet flow rate parameters were considered void and not used within the context of this study. Quantitative analysis of all air contaminant samples took place at MPL Laboratories (Perth, Western Australia), an environmental chemistry laboratory accredited for chemical testing with the National Association of Testing Authorities (NATA). Airborne samples for dust were analysed according to AS 2985:2009 for Respirable Dust and AS 3640:2009 for Inhalable Dust, which report the difference between the initial and final weight of the sample filter. Respirable crystalline silica was measured after ashing, redeposition and Fourier-transform infrared spectroscopy (FTIR) determination. Point estimate values of (i) lowest, (ii) highest, and (iii) most common (mode) were calculated from the data set in order to define the true nature of the respective exposure profiles. Descriptive statistics for all measured data can be found in the S1 File.

**Table 1:** Personal samples (measured data) collected by contaminant for each job role.

Contaminant	Job role			
	Project driller	Mobile equipment operator	Fixed plant maintainer	Drill and blast operator
Respirable crystalline silica	*n = 220*	*n = 310*	*n = 200*	*n = 210*
Respirable dust	*n = 220*	*n = 310*	*n = 200*	*n = 210*
Inhalable dust	*n = 300*	*n = 350*	*n = 330*	*n = 280*

### Statistical encoding of elicitations

The majority of the elicited values were strongly left or right skewed, e.g., the most common value was equal to the minimum or maximum elicited value. A previous study showed that the scaled Beta distribution provided a better fit than the normal and lognormal distributions, particularly for strongly skewed data [30]. Therefore, for each expert, a scaled Beta distribution was fitted to each job role and contaminant combination by scaling the elicited values to the range [0, 1] [30]. A least squares approach was used to estimate the α and β parameters of the Beta distribution by ensuring that the distance between the elicited and encoded quantities was minimised using mean sum of squares (MSS) [30, 34, 35]. The expert’s mode (most common) was defined as (α – 1)/(α + β – 2). When the expert’s lowest and most common estimate values were the same, then α was set to one and least squares was applied to identify β parameter [30]. Similarly, when the highest and most common estimate values were the same, then β was set to one and α was estimated using least squares. The function ‘optim’ in R [36] was employed to search across the parameter space to identify the best α and β parameters that minimise MSS [37]. To estimate a single distribution which captures the combined experts’ values, we applied linear pooling by calculating the sum of the individual expert’s distributions [21, 30].

The measured data were also encoded into scaled Beta distributions. The mode and the lower and upper bounds for the 95% confidence interval were calculated for each job role and contaminant measured data combination. These summary statistic values were then encoded into scaled Beta distributions using the same methodology as the elicited values.

## Results

**Fig 1:**
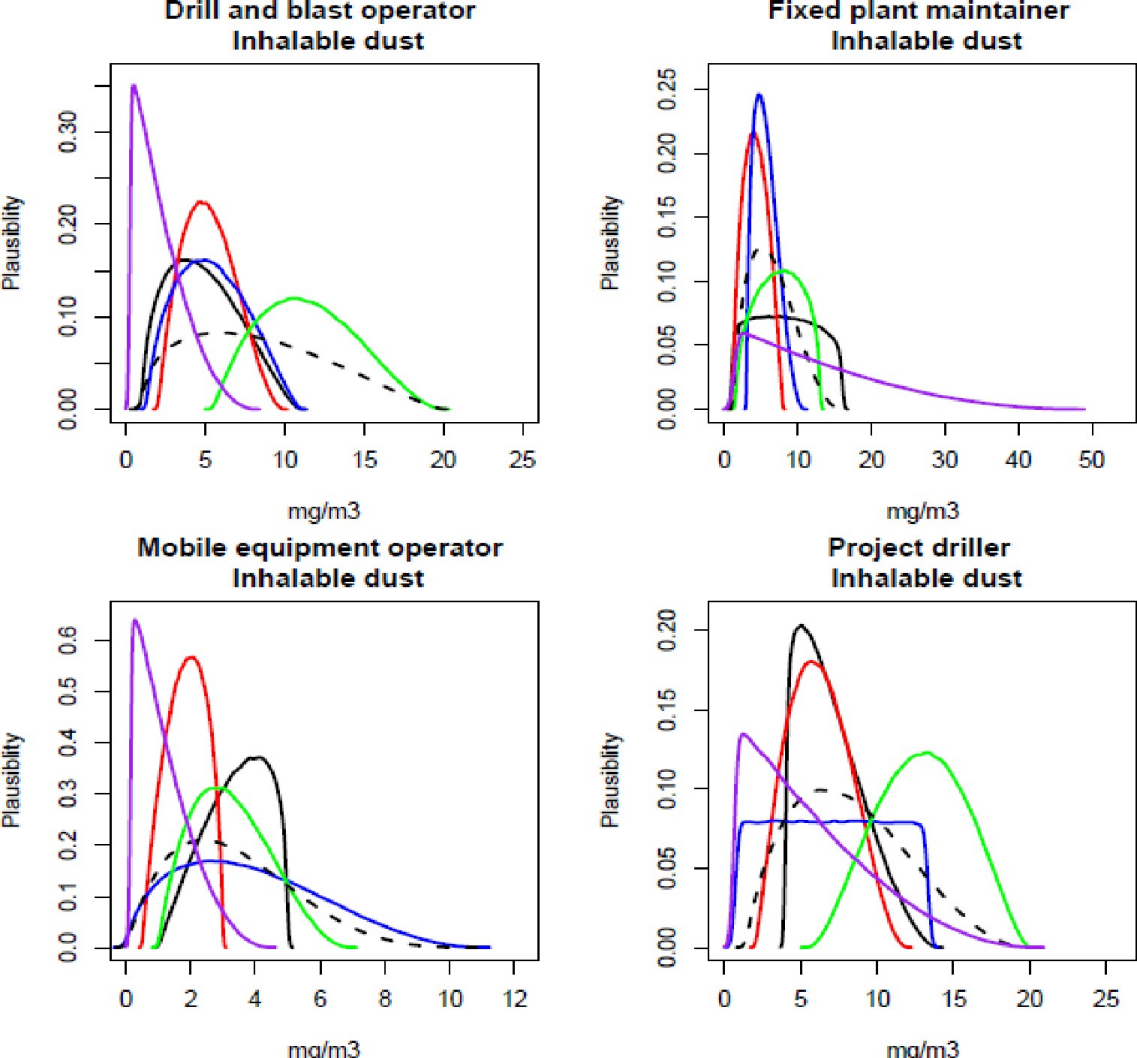
Expert estimates and measured data of inhalable dust concentrations. Each curve depicts the experts support (probability density) or measured data encoded into a scaled Beta distribution. Experts are denoted in the colours blue, red, black and green; combined experts are the dashed line. Measured data is presented as purple.

**Fig 2:**
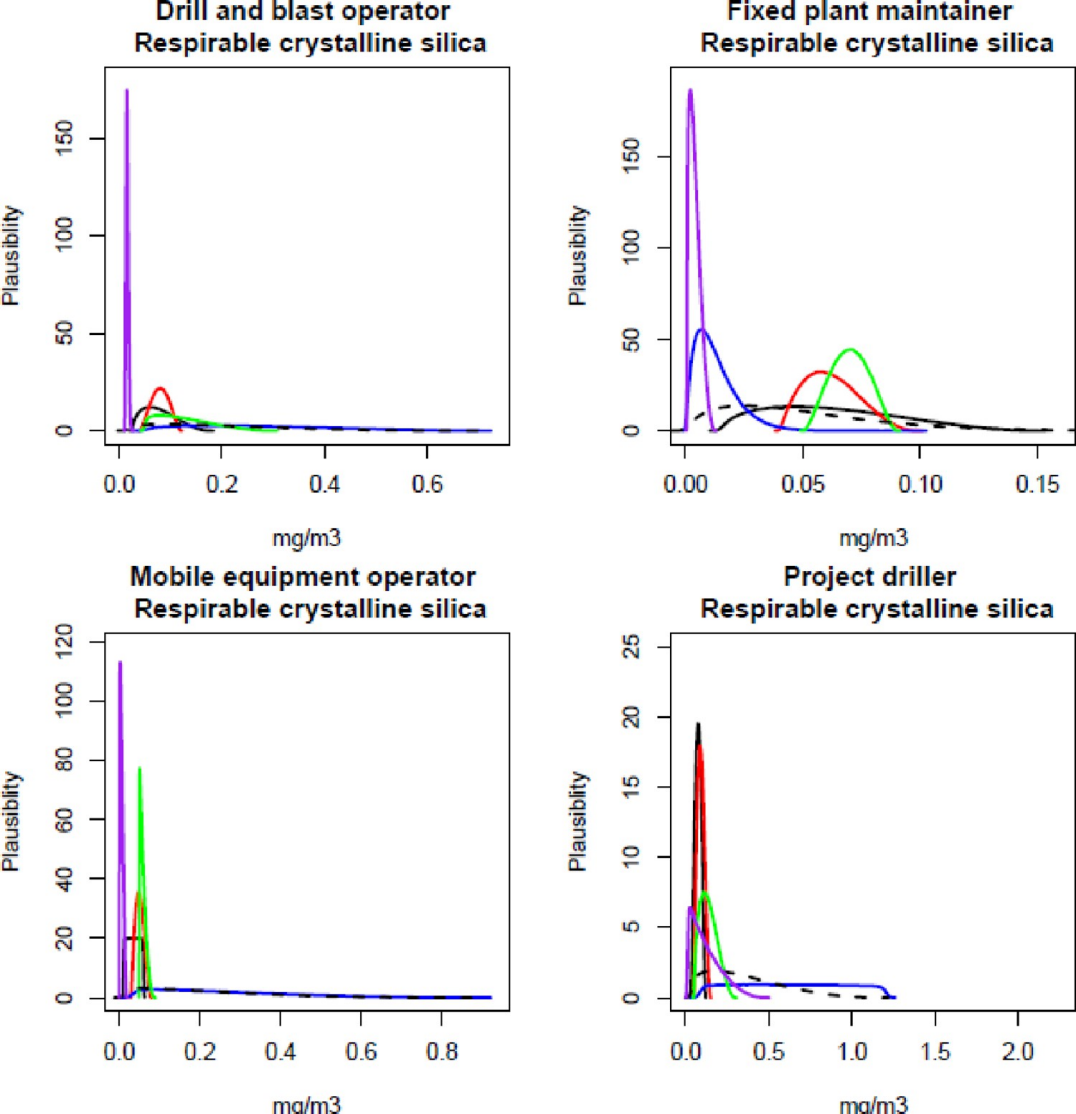
Expert estimates and measured data of respirable crystalline silica concentrations. Each curve depicts the experts support (probability density) or measured data encoded into a scaled Beta distribution. Experts are denoted in the colours blue, red, black and green; combined experts are the dashed line. Measured data is presented as purple.

**Fig 3:**
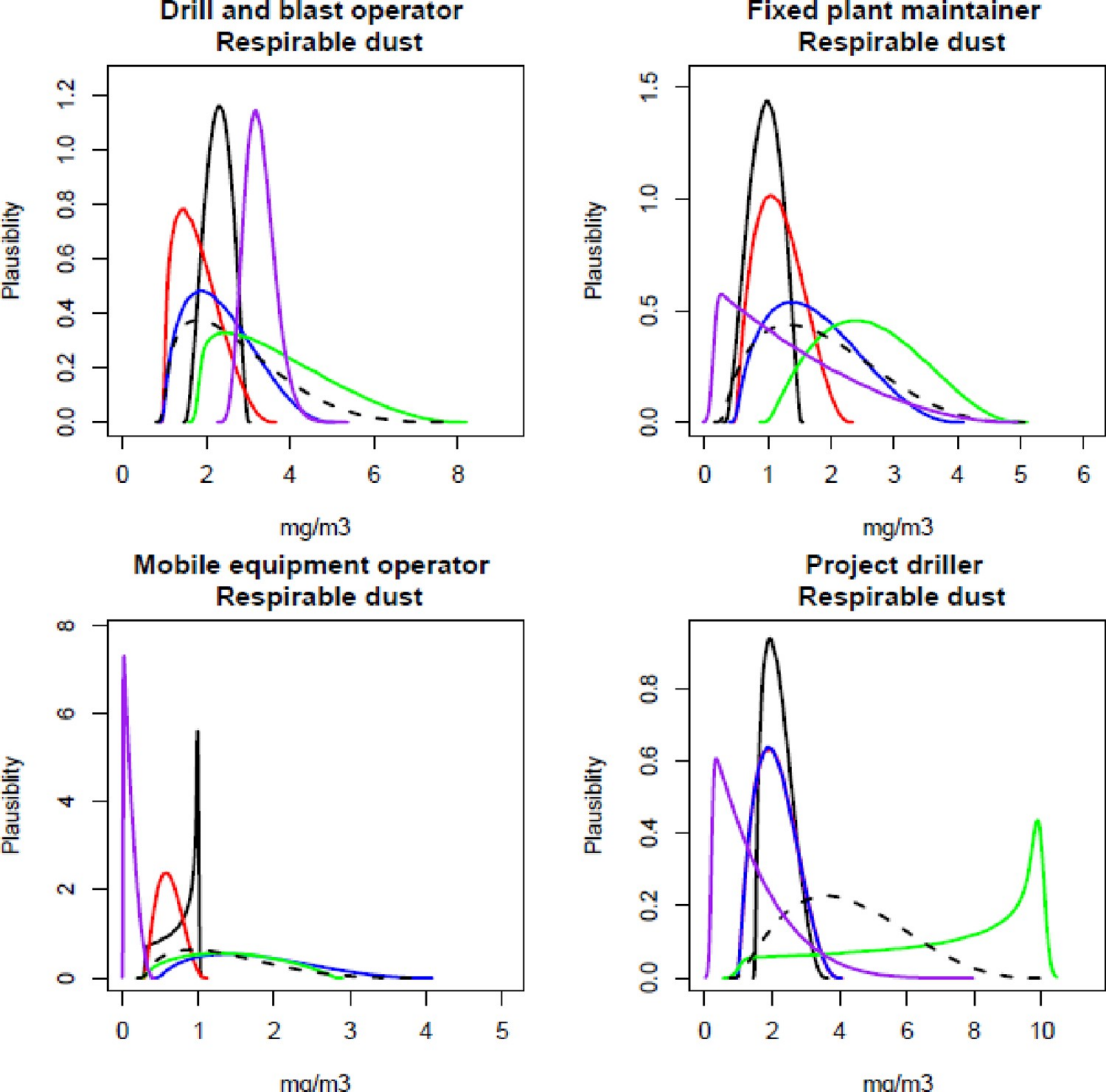
Expert estimates and measured data of respirable dust concentrations. Each curve depicts the experts support (probability density) or measured data encoded into a scaled Beta distribution. Experts are denoted in the colours blue, red, black and green; combined experts are the dashed line. Measured data is presented as purple.

**Fig 4:**
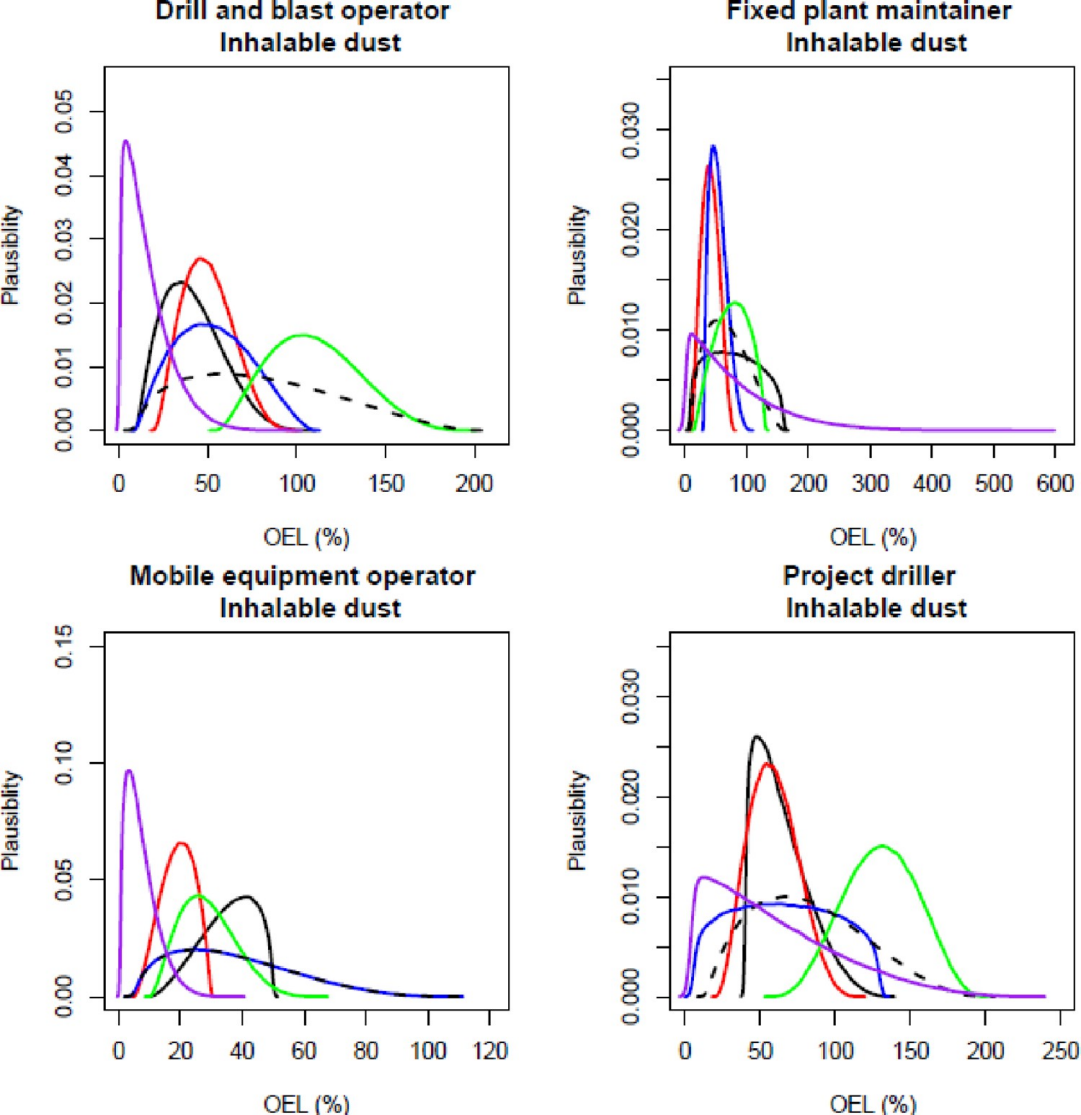
Expert estimates and measured data of inhalable dust percentage of occupational exposure limit (OEL). Each curve depicts the experts support (probability density) or measured data encoded into a scaled Beta distribution. Experts are denoted in the colours blue, red, black and green; combined experts are the dashed line. Measured data is presented as purple.

**Fig 5:**
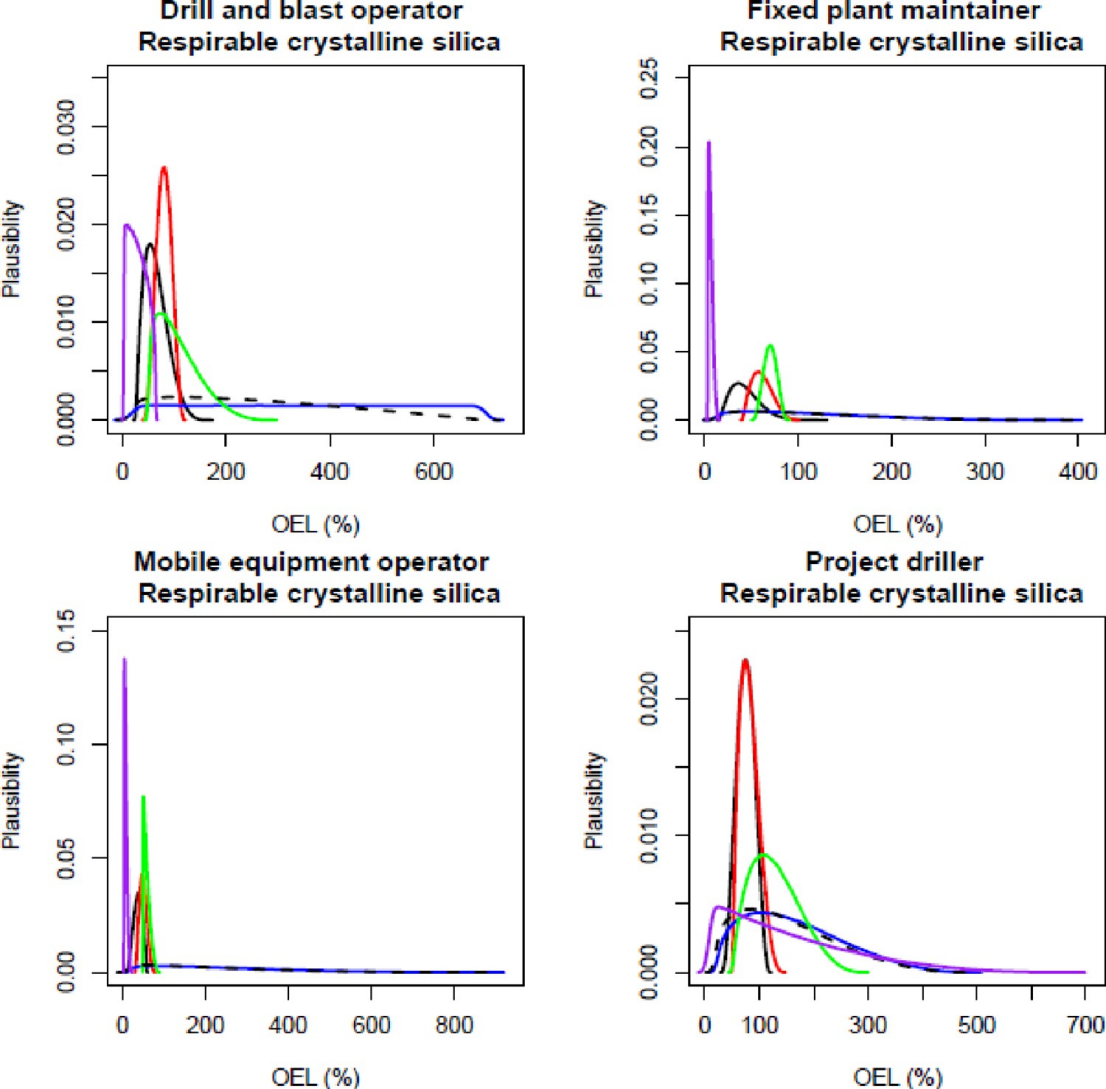
Expert estimates and measured data of respirable crystalline silica percentage of occupational exposure limit (OEL). Each curve depicts the experts support (probability density) or measured data encoded into a scaled Beta distribution. Experts are denoted in the colours blue, red, black and green; combined experts are the dashed line. Measured data is presented as purple.

**Fig 6:**
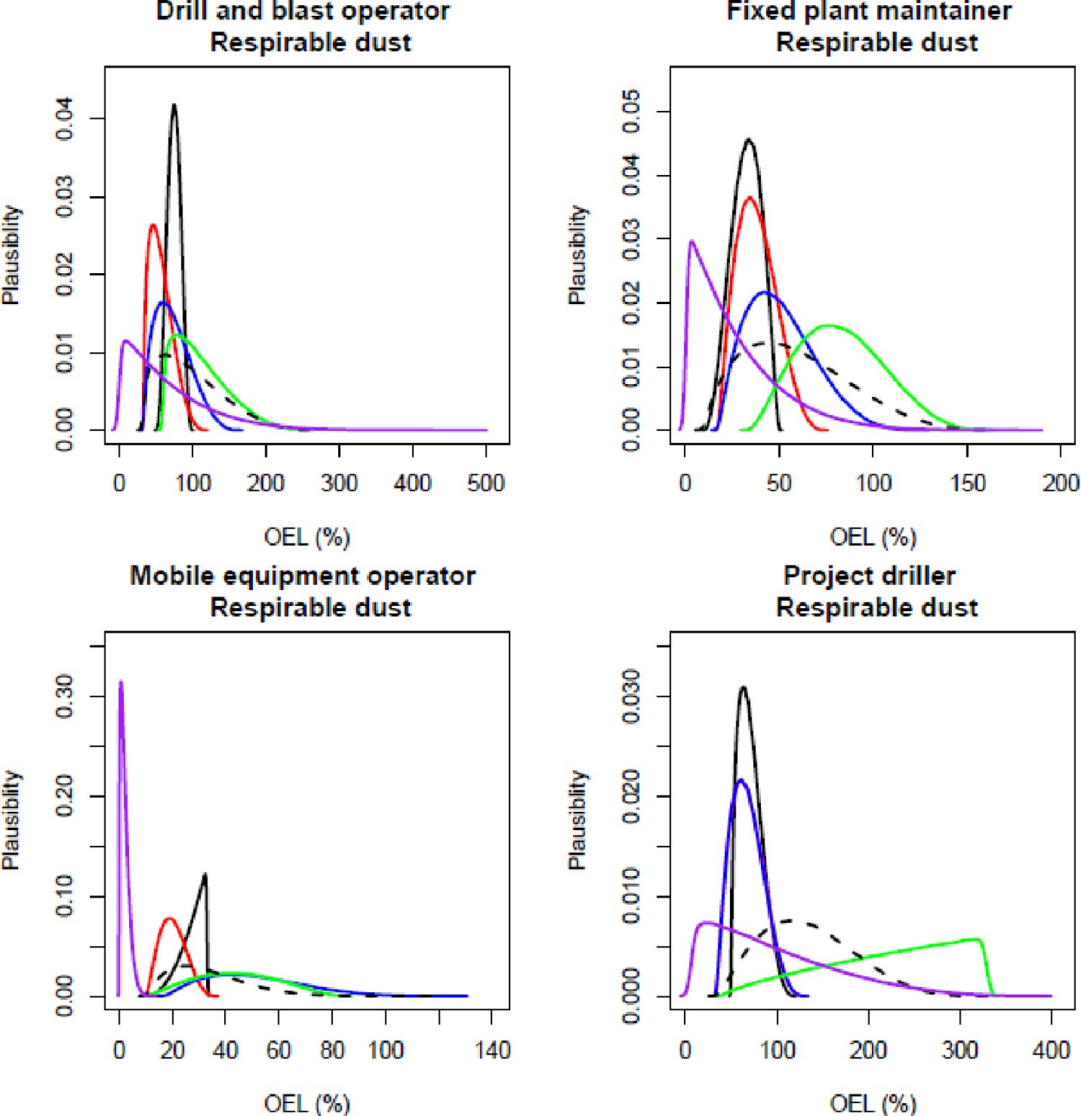
Expert estimates and measured data of respirable dust percentage of occupational exposure limit (OEL). Each curve depicts the experts support (probability density) or measured data encoded into a scaled Beta distribution. Experts are denoted in the colours blue, red, black and green; combined experts are the dashed line. Measured data is presented as purple.

The participating experts reported a timeframe of between 45–60 minutes to complete all elicitations (all job roles, all contaminants), and all experts expressed confidence that the process captured their knowledge of exposure. Figs 1–3 show the individual and combined expert plausible (density) estimates of exposure concentration (mg/m^3^) compared with the measured data across the four job roles with respect to each contaminant and Figs 4–6 show values in percentage of the relevant OEL. The term ‘plausibility’ can be defined as the degree of expert support on the estimates of exposure concentration and OEL estimates [30]. Most measured data follow a lognormal distribution, exhibiting right (positive) skewness [38], and this is observed in 60% of the measured data distributions (all Figs except 2 and 5). Within all Figs, the experts are denoted in the colours blue, red, black and green. The combined expert’s distribution is denoted with a dashed line and measured data is presented as a purple line.

Comparison of the most common exposure value between the experts and the measured data demonstrate that all experts provided a value higher than the measured value for all contaminants and all job roles, meaning exposure has been overestimated for both percentage of the OEL and concentration in all elicitations. For the highest exposure value, the experts overestimated exposure 41% and 54% of the time respectively for OEL and concentration. For the lowest exposure values experts overestimated exposure 96% of the time for both OEL and concentration when compared with the measured data.

For inhalable dust concentration, all four experts were similar to the measured data distributions for the job roles of fixed plant maintainer and mobile equipment operator (Fig 1). However, for the other two roles, the green expert estimated higher values than the other experts and the measured data.

For all four respirable crystalline silica plots, the measured data had very tight distributions (Fig 2). The blue expert’s distribution was very wide compared to measured data and all other experts’ distributions. For the job role drill and blast operator, all expert’s most common values were higher than the measured distribution. For fixed plant maintainer, the blue expert was lower and most common values agreed with the measured data; however, the other three (black, red and green) expert’s lower and most common values were higher than the measured data.

For respirable dust concentration, no expert agreed with the measured data, and the range of blue and green experts’ distribution was similar (Fig 3). The green expert’s distribution was very different to the measured data and all other experts’ distributions for the job role project driller.

For the estimates of the percent of the inhalable dust OEL, all expert distributions fell within the range of the measured data (Fig 4). In addition, all expert distributions were similar to the measured data for the job role of fixed plant maintainer. For the other job roles, the modes (most common value) of the expert distributions were higher than the measured data. All estimates of the most common value were similar to the measured data for the job role of project driller when assessing the percent of the OEL for respirable crystalline silica (Fig 5). For the other three job roles, the blue expert distribution had a wide range when compared to the measured data and other experts.

For the assessment of the percent of the respirable dust OEL, the measured data distribution were right skewed except for the job role of mobile equipment operator (Fig 6). The green expert’s distributions disagreed with the measured data in all four job roles. All lowest elicited values were in the range of the measured data. For drill and blast operator, all experts had a similar distribution compared with the measured distribution, however the most common value of all the experts was slightly higher compared to the mode of the measured data.

## Discussion

The main purpose of this study was to use expert elicitation to assess the professional judgement of a group of occupational hygienists. We have presented and evaluated a statistical methodology for the encoding of elicited information into distributions from multiple experts. We applied a scaled Beta distribution to expert and measured data; this approach was able to accommodate both left and right skewed distributions as well as “normal” distributions. Our findings suggest that the participating occupational hygienists within this study were inclined to overestimate exposures and that they were more accurate at estimating percentage of OEL than concentration values (refer to study comparison tables in the S2 Data). Our approach differs from previous research in the way in which exposure assumptions were elicited, by focusing on contaminant concentration and attribution of an exposure standard percentage estimate.

The use of expert knowledge in decision making has been gaining traction in many scientific disciplines, most notably in areas where a traditional approach of utilising observed data may not be a practical option [19–21]. Most assessments conducted within a comprehensive exposure assessment program are qualitative, that is, completed without measured data. This approach is by design and is practically necessary, as the number of exposure scenarios in a workplace may total in the hundreds in which conducting quantitative exposure assessments (i.e., using measured data with sufficient samples to support decision making) for every scenario is not feasible [2]. For example, the American Industrial Hygiene Association (AIHA) exposure assessment strategy calls for initial, qualitative assessments of exposures, relative to a reference exposure level [15].

Occupational hygienists review the workforce, materials, exposure agents, tasks, equipment, exposure controls and identify exposure groups that will be assessed and controlled depending on the final judgments. The exposure evaluation for any job role requires the selection of an OEL and a judgment by the hygienist about where the decision statistic (for example, the 95th percentile of the exposure distribution for the job role) falls in relation to the OEL [15]. Professional judgement is considered a ‘tool in the toolkit’ of the hygienist and serves as a key factor when making a determination on whether an exposure is acceptable in the context of an occupational environment. However, for the most part, subjective qualitative judgments in the field of occupational hygiene have proven to be no more accurate than random chance. This may be because patterns of exposures in many workplaces have a significant degree of uncertainty and unpredictability and there may be little or no data available on these exposure levels. Such situations have been defined as ‘low-validity’ environments [22] and perhaps somewhat paradoxically, judgement decisions have been shown to be most accurate in these highly uncertain situations, particularly when paired with checklists or models. The use of a checklist that considers consistent inputs is shown to be more reliable at arriving at a judgement than a purely ‘human’ focussed way but this has not previously been assessed in the occupational hygiene setting [4, 12, 22].

A key observation from this study is the experts’ proclivity to consistently overestimate exposures. This appears to be a point of difference when compared to similar studies where there was a significant underestimation bias in the exposure judgments when the range is examined [3, 4, 12]. The reasons behind this finding are worth exploring. In other expert elicitation studies [19–21] experts are typically able to estimate the range of measured data distribution quite accurately, however the most common value tends to be higher than the measured value. Our study found that the most common exposure value between the experts and the measured data was higher than the measured value for all contaminants and all job roles for both percentage of the OEL and concentration in all elicitations. We found that the experts lowest exposure value was nearly always (96% of the time) higher than that of the measured equivalent and the highest exposure value was overestimated about half of the time (41% and 54% of the time for percentage of OEL and concentration respectively). These findings suggest that hygienists may be more concerned about the upper bound of an exposure profile as opposed to the lower and therefore concentrated more on estimating this more carefully.

Comparing the expert versus the measured data distributions show that the experts appear to be able to estimate percentage of the OEL more accurately than concentration. This may be attributable to a variety of factors, including risk communication. Given one of the mandates of the occupational hygienist is to ‘distil’ complex data into easy-to-understand messages for a workforce, many hygienists have taken to expressing results of monitoring data as percentages of the applicable exposure standard and so this way to present data is likely to be more familiar to them.

With respect to the experts, the green expert was notably divergent from the measured data and their elicitations often yielded different results from the other experts. This disparity warrants further investigation into how the green expert executed the elicitations, and whether any cognitive biases attributable to the heuristics of availability, representativeness, and anchoring and adjustment were present during this exercise. A deeper dive into the determinants of the elicited values would provide transparency around the decision-making practices of each expert.

A strength of the study was the statistical encoding of both expert and measured data into scaled Beta distributions. The advantage of the scaled Beta distribution when compared with the normal and lognormal distributions is that it performs better over all levels of skewness, in particular providing accurate encoded values under extreme skewness [30]. This is particularly useful when the skewness is expected to be high, or in situations where the degree of uncertainty is high. Both situations are present within the context of this study, and this illustrates why probabilistic methods are attractive to hygienists who are required to make exposure judgments with limited sampling data [39].

A further strength of this study was that we had a large amount of measured data to use for comparison against the expert elicitations. A standard approach to exposure assessment in the field of occupational hygiene dictates randomly sampling 6–10 events of a specific job role and calculating an upper tail decision statistic such as the 95th percentile with an upper confidence limit (e.g. 90th or 95th) [15]. This approach to exposure assessment has been utilised in the field for many years and was based on the assumption of a stable and predictable work environment wherein a reliable mean and geometric standard deviation can be calculated after 6–10 samples [15]. With the advent of a more dynamic workforce expected to complete multiple tasks across different work environments (as is the case in the mining industry), the concept of full-shift personal monitoring to define the exposure profile of a job role or similar exposure group (SEG) may not be an optimal approach. Given this, the large dataset in this study was useful in capturing the real distribution of the measured data that may be present in a dynamic work environment [40]. With the introduction of sensor measurement technology (sometimes referred to as ‘real-time’ monitoring) future studies may focus on comparisons between experts and quantitative measurements that are task or source based, which may present a more accurate picture of a worker’s exposure in a dynamic occupational environment.

A potential limitation of this study was the number of experts recruited for elicitation. Although there is no absolute guideline on which to base the number of experts invited to provide input, a panel of expert elicitation practitioners determined that at least six experts should be included to ensure robustness of results [41]. The same panel also concluded that a point of diminishing returns was reached beyond twelve experts. Future studies may wish to expand the number of experts involved to further broaden the range of experiences that contribute to a person’s professional judgement. However, a challenge to these further studies is the availability of both general and industry-specific experts. In addition, the study was completed in the context of a mining environment with only three agents of interest, all of which were particulates. Future studies should ensure a larger sample size of experts are recruited and assessment be focused to a larger suite of airborne contaminants across other industries.

Another limitation of the study are the uncontrolled conditions that the expert elicitations were completed. The elicitation steps, parameter descriptors, elicitation tool (Excel document) and relevant exposure limits were provided to the experts by email; however, the authors were not aware, and did not specifically enquire, as to any additional resources or information used by the experts when completing their judgements. In addition, a ‘hard’ timeframe for return of the elicitation tool with completed judgements was not set by the authors, rather a ‘request’ was made to return the completed protocol document within a two-week period. Further studies should ensure that any additional resources or information utilised during the elicitation process are categorised and reported. Given the role of a practicing hygienist, it may be impractical to expect elicitations be completed under controlled conditions (i.e., in a supervised exam room), however specifying a set timeframe for completion of the elicitation protocol should also be considered.

## Conclusions

The results in this study suggest that, in the absence of measured data and under the same methodology described within this paper, the participating occupational hygienists tended toward an overestimation of exposures. The practical implication of overestimating may be an ‘overprotection’ of workgroups, or a misallocation of resources such as risk controls, respiratory protection, health surveillance and awareness programs. Conversely, the consequences of underestimating exposure (as has been reported in other studies) may leave workers unprotected.

From a practitioner standpoint, hygienists would err toward a more conservative approach to protecting worker health if given the choice; however, there are pros and cons to this. For example, a conservative approach may result in higher order respiratory protection being prescribed in the absence of actual risk, which may impact adversely on an individual’s metabolic load. In a high heat environment, the result of this could be dangerous to the individual through the development of a heat-related illness. Similarly, overestimation may result in scant resources not being adequately apportioned based on risk, which could extend out to critical health surveillance (i.e., disease identification) services.

Despite these findings, it is clear that the field of occupational hygiene is integral to the global effort of protecting worker health. The elicitation protocol used in this study, although reflective of ‘real world’ challenges of assessing exposures in the absence of measured data, was designed to require a high degree of specificity when the experts were making their respective judgements. The concept of exposure assessment is complex, with the amount of information required to be assessed often exceeding the capacity of the pre-frontal cortex, the decision-making area of the brain [2, 42]. This overload can make the brain vulnerable to flaws of memory and distraction, which can lead to bias and over-confidence in decision-making [2, 42].

These findings suggest that improved accuracy in exposure assessment in the absence of measured data is needed, particularly in the context of a dynamic work environment where job roles are expected to complete tasks across different work fronts, as is the case within an Australian mining context. Further efforts should assess the expert’s decision-making process and the determinants of their judgements. Future research should focus on these determinants of professional judgement to better assess accuracy and inform formalised training programmes, models, and other tools to improve exposure assessment within the discipline of occupational hygiene.

## Supporting information

S1 Data (XLSX)

S2 Data (XLSX)

S1 File (DOCX)
