# Supplementary material for: Use of expert elicitation in the field of occupational hygiene: Comparison of expert and observed data distributions
Source: PLoS One. 2022 Jun 8;17(6):e0269704. doi: 10.1371/journal.pone.0269704 (PMC9176777; doi:10.1371/journal.pone.0269704)
Supplement: S1 File — (DOCX) [file pone.0269704.s003.docx]

**Descriptive statistics from measured data**

Drill & Blast Operator, Respirable Crystalline Silica

| **DESCRIPTIVE STATISTICS** |  |
| --- | --- |
| Maximum (max) | 118.73 |
| Minimum (min) | 2.64 |
| Range | 116 |
| Percent above OEL (%>OEL) | 0.0 |
| Mean | 12 |
| Median | 8 |
| Standard deviation (s) | 13 |
| Mean of log transformed data (LN) | 2.18 |
| Std. deviation of log transformed data(LN) | 0.596 |
| Geometric mean (GM) | 8.9 |
| Geometric standard deviation (GSD) | 1.81 |

Project Driller, Respirable Crystalline Silica

| **DESCRIPTIVE STATISTICS** |  |
| --- | --- |
| Maximum (max) | 690.84998 |
| Minimum (min) | 3.52 |
| Range | 687 |
| Percent above OEL (%>OEL) | 8.2 |
| Mean | 45 |
| Median | 16 |
| Standard deviation (s) | 105 |
| Mean of log transformed data (LN) | 2.91 |
| Std. deviation of log transformed data(LN) | 1.195 |
| Geometric mean (GM) | 18.3 |
| Geometric standard deviation (GSD) | 3.30 |

Mobile Equipment Operator, Respirable Crystalline Silica

| **DESCRIPTIVE STATISTICS** |  |
| --- | --- |
| Maximum (max) | 23.25 |
| Minimum (min) | 2.64 |
| Range | 21 |
| Percent above OEL (%>OEL) | 0.0 |
| Mean | 7 |
| Median | 6 |
| Standard deviation (s) | 4 |
| Mean of log transformed data (LN) | 1.84 |
| Std. deviation of log transformed data(LN) | 0.331 |
| Geometric mean (GM) | 6.3 |
| Geometric standard deviation (GSD) | 1.39 |

Fixed Plant Maintainer, Respirable Crystalline Silica

| **DESCRIPTIVE STATISTICS** |  |
| --- | --- |
| Maximum (max) | 20.969999 |
| Minimum (min) | 2.99 |
| Range | 18 |
| Percent above OEL (%>OEL) | 0.0 |
| Mean | 7 |
| Median | 6 |
| Standard deviation (s) | 3 |
| Mean of log transformed data (LN) | 1.81 |
| Std. deviation of log transformed data(LN) | 0.287 |
| Geometric mean (GM) | 6.1 |
| Geometric standard deviation (GSD) | 1.33 |

Project Driller, Inhalable Dust

| **DESCRIPTIVE STATISTICS** |  |
| --- | --- |
| Maximum (max) | 235.52 |
| Minimum (min) | 3.48 |
| Range | 232 |
| Percent above OEL (%>OEL) | 5.0 |
| Mean | 25 |
| Median | 12 |
| Standard deviation (s) | 50 |
| Mean of log transformed data (LN) | 2.56 |
| Std. deviation of log transformed data(LN) | 0.919 |
| Geometric mean (GM) | 12.9 |
| Geometric standard deviation (GSD) | 2.51 |

Mobile Equipment Operator, Inhalable Dust

| **DESCRIPTIVE STATISTICS** |  |
| --- | --- |
| Maximum (max) | 84.629997 |
| Minimum (min) | 0.19 |
| Range | 84 |
| Percent above OEL (%>OEL) | 0.0 |
| Mean | 8 |
| Median | 4 |
| Standard deviation (s) | 13 |
| Mean of log transformed data (LN) | 1.30 |
| Std. deviation of log transformed data(LN) | 1.126 |
| Geometric mean (GM) | 3.7 |
| Geometric standard deviation (GSD) | 3.08 |

Fixed Plant Maintainer, Inhalable Dust

| **DESCRIPTIVE STATISTICS** |  |
| --- | --- |
| Maximum (max) | 675.77002 |
| Minimum (min) | 0.41 |
| Range | 675 |
| Percent above OEL (%>OEL) | 3.4 |
| Mean | 37 |
| Median | 9 |
| Standard deviation (s) | 74 |
| Mean of log transformed data (LN) | 2.45 |
| Std. deviation of log transformed data(LN) | 1.293 |
| Geometric mean (GM) | 11.6 |
| Geometric standard deviation (GSD) | 3.64 |

Drill & Blast Operator, Inhalable Dust

| **DESCRIPTIVE STATISTICS** |  |
| --- | --- |
| Maximum (max) | 352.17001 |
| Minimum (min) | 0.51 |
| Range | 352 |
| Percent above OEL (%>OEL) | 2.1 |
| Mean | 28 |
| Median | 13 |
| Standard deviation (s) | 55 |
| Mean of log transformed data (LN) | 2.48 |
| Std. deviation of log transformed data(LN) | 1.120 |
| Geometric mean (GM) | 12.0 |
| Geometric standard deviation (GSD) | 3.06 |

Project Driller, Respirable Dust

| **DESCRIPTIVE STATISTICS** |  |
| --- | --- |
| Maximum (max) | 395.39999 |
| Minimum (min) | 2.67 |
| Range | 393 |
| Percent above OEL (%>OEL) | 2.2 |
| Mean | 23 |
| Median | 10 |
| Standard deviation (s) | 58 |
| Mean of log transformed data (LN) | 2.45 |
| Std. deviation of log transformed data(LN) | 0.942 |
| Geometric mean (GM) | 11.6 |
| Geometric standard deviation (GSD) | 2.57 |

Mobile Equipment Operator, Respirable Dust

| **DESCRIPTIVE STATISTICS** |  |
| --- | --- |
| Maximum (max) | 68.889999 |
| Minimum (min) | 0.22 |
| Range | 69 |
| Percent above OEL (%>OEL) | 0.0 |
| Mean | 7 |
| Median | 4 |
| Standard deviation (s) | 10 |
| Mean of log transformed data (LN) | 1.34 |
| Std. deviation of log transformed data(LN) | 0.834 |
| Geometric mean (GM) | 3.8 |
| Geometric standard deviation (GSD) | 2.30 |

Fixed Plant Maintainer, Respirable Dust

| **DESCRIPTIVE STATISTICS** |  |
| --- | --- |
| Maximum (max) | 225.92 |
| Minimum (min) | 0.32 |
| Range | 226 |
| Percent above OEL (%>OEL) | 1.6 |
| Mean | 23 |
| Median | 15 |
| Standard deviation (s) | 28 |
| Mean of log transformed data (LN) | 2.46 |
| Std. deviation of log transformed data(LN) | 1.208 |
| Geometric mean (GM) | 11.7 |
| Geometric standard deviation (GSD) | 3.35 |

Drill & Blast Operator, Respirable Dust

| **DESCRIPTIVE STATISTICS** |  |
| --- | --- |
| Maximum (max) | 98.470001 |
| Minimum (min) | 0.02 |
| Range | 98 |
| Percent above OEL (%>OEL) | 0.0 |
| Mean | 13 |
| Median | 11 |
| Standard deviation (s) | 14 |
| Mean of log transformed data (LN) | 2.13 |
| Std. deviation of log transformed data(LN) | 1.141 |
| Geometric mean (GM) | 8.4 |
| Geometric standard deviation (GSD) | 3.13 |
